# Executive control and working memory are involved in sub-second repetitive motor timing

**DOI:** 10.1007/s00221-016-4839-6

**Published:** 2016-11-24

**Authors:** Linus Holm, Olympia Karampela, Fredrik Ullén, Guy Madison

**Affiliations:** 1 Department of Psychology, Umeå University, 901 87 Umeå, Sweden; 2 Department of Neuroscience, Karolinska Institutet, 171 77 Stockholm, Sweden

**Keywords:** Timing, Dual task, Executive functions, Working memory, Continuation tapping, Isochronous interval production, Random action generation

## Abstract

The nature of the relationship between timing and cognition remains poorly understood. Cognitive control is known to be involved in discrete timing tasks involving durations above 1 s, but has not yet been demonstrated for repetitive motor timing below 1 s. We examined the latter in two continuation tapping experiments, by varying the cognitive load in a concurrent task. In Experiment 1, participants repeated a fixed three finger sequence (low executive load) or a pseudorandom sequence (high load) with either 524-, 733-, 1024- or 1431-ms inter-onset intervals (IOIs). High load increased timing variability for 524 and 733-ms IOIs but not for the longer IOIs. Experiment 2 attempted to replicate this finding for a concurrent memory task. Participants retained three letters (low working memory load) or seven letters (high load) while producing intervals (524- and 733-ms IOIs) with a drum stick. High load increased timing variability for both IOIs. Taken together, the experiments demonstrate that cognitive control processes influence sub-second repetitive motor timing.

## Introduction

Consider a simple rhythmic motor task such as tapping at a regular pulse with a finger. Clearly, this task is under voluntary control in the sense that we can initiate, terminate or modify the behavior––e.g., change the force or pace of the taps––by will. However, this does not imply that cognitive control is also needed for performance itself, i.e., the generation of precisely timed finger taps. There is a long established association between cognitive performance (e.g., intelligence, *g*) and timing in general (i.e., chronometric tasks such as reaction time, inspection time, time discrimination and interval production), but the nature of this relationship is poorly understood (see, e.g., Deary et al. 2001; Galton 1883; Jensen 2006; Silverman 2012; Troche and Rammsayer 2011; Ullén et al. 2012, 2015). To elucidate the relationship between cognition and timing, experiments are needed that directly test the causal relationship between cognitive functions such as cognitive control and timing performance.

In the present study, we use “cognitive control” to denote the collective functions of active maintenance and manipulation of information, which corresponds to working memory and the executive functions, respectively (Baddeley 1992; Fuster 2000). While this is an important functional distinction, these two broad functions appear to recruit highly overlapping neural circuitry (Veltman et al. 2003), suggesting a superordinate cognitive control network in the brain (Eriksson et al. 2015; Niendam et al. 2012). For instance, parametric variation of “working memory” load, such as varying the number of letters retained in a Sternberg task, and parametric variation in “executive load” by varying the *n* in an *n*-back task have almost identical effects on brain activation (Veltman et al. 2003).

 Repetitive motor timing is critically important for many daily––and perhaps uniquely human––activities such as dancing and music production, and the ability to synchronize activities with other individuals. Other primates, such as macaque monkeys, display similar timing abilities as do humans, but with some important differences (Zarco et al. 2009). Specifically, monkeys exhibit higher timing variability than humans in unsupported repetitive timing, such as tapping out an isochronous sequence with the fingers in the absence of a metronome or other rhythmic input. While there are many differences between man and monkey, fronto-parietal brain volume and the cognitive control it provides are often hailed as an important difference (see, e.g., Mantini et al. 2013; Passingham 2009). One possibility is thus that interspecies performance differences in unsupported timing reside in differences in cognitive control.

Available data suggest that the involvement of cognitive control in timing varies with the timed duration. In a range from approximately 350 ms to 1 s, perceptual discrimination and continuation tapping variability are virtually linear with time (Getty 1975; Ivry and Hazeltine 1995; Michon 1964; Zarco et al. 2009). Above 1 s, the relationship between interval duration and performance exhibits several nonlinearities that are seen as breakpoints in the relation between the two variables (Madison 2001, 2004; for a proposed explanation, see Madison and Delignières 2009). Indeed, many timing studies endorse a distinction between “automatic” timing of intervals below about 1 s and “cognitive” timing of longer durations (Karmarkar and Buonomano 2007; Lewis and Miall 2003, 2006; Maes et al. 2015; Rammsayer 1999; Rammsayer and Troche 2014). Timing may thus be supported by more or less different mechanisms above and below about 1 s, although there are also other breakpoints in the performance–IOI relationship that might be related to functional differences. The dorsolateral prefrontal cortex, a central brain region for cognitive control (Jahanshahi et al. 2000; Owen et al. 2005), increases its activity when interval duration increases (Lewis and Miall 2006). In line with this, Brown (1997) found that the difficulty level in a math problem affected timing precision in a concurrent 2-s interval production task. Other studies support that cognitive control functions are involved in the timing of long durations spanning several seconds and minutes (Brown 2006; Logie et al. 2004; Marsh and Hicks 1998).

It remains unclear, however, whether the same is true for voluntary timing in the sub-second range. Some support for cognitive control involvement in sub-second timing has come from studies using dual-tasks paradigms (Kee et al. 1986; Hiscock et al. 1989; McFarland and Ashton 1978; Sergent et al. 1993). The general logic of this paradigm is that if two tasks recruit the same limited cognitive resource, performance in either or both tasks should suffer when performed together. Kee et al. (1986) reported that solving anagrams and thinking out loud while concurrently producing movements every 380 ms resulted in more variable performance than single-task conditions. Another study (McFarland and Ashton 1978) found that increased numbers of intervening items in a running recognition memory task increased variability of simultaneous finger tapping.

On the other hand, both (Michon 1966) and (Nagasaki 1990) report relatively marginal dual-task effects on finger tapping. Further indications that cognitive control is of limited importance for motor timing come from studies showing that responses to distractors are unconscious and involuntary (Repp 2006) and that subliminal perturbations affect tapping performance (Madison and Merker 2004). Moreover, a dual-task study involving finger tapping found no significant effect of cognitive control on repetitive motor timing variability in the range from 0.5 to 2.0 s (Holm et al. 2013). However, high cognitive load conditions in that study did generally generate higher timing variability than did low load conditions, and the statistical power may have been insufficient to reliably detect the effect on sub-second timing. Finally, motor timing appears to be supplied by a rather distributed cortico-striatal network in the brain (see, e.g., Buhusi and Meck 2005; Merchant et al. 2013 for reviews). Therefore, cognitive control might reasonably only partly explain the management of timed activities by humans.

To summarize, the earlier literature clearly suggests that cognitive control is important for long duration timing, but it is unclear whether cognitive control also has some influences on sub-second motor timing. The present study directly examined the influence of cognitive control on sub-second timing by employing the synchronization-continuation task (Stevens 1886; Wing and Kristofferson 1973). Participants produced regular time intervals by means of key presses on a synthesizer keyboard (Experiment 1) or by beating a drumstick against a drum pad (Experiment 2), initially in phase with an isochronous metronome, and thereafter producing self-paced beats without a metronome. Variability was analyzed for the inter-beat intervals during the continuation phase. In Experiment 1, we addressed the influence of executive functions on timing variability. Experiment 2 complemented this by investigating the influence of working memory on timing variability. The study thus allows for potentially distinguishing between executive function involvement and working memory influence on repetitive timing. Specifically, does unsupported repetitive motor timing recruit the active handling aspects provided by the executive functions or the maintenance aspect as provided by working memory? Or is repetitive motor timing independent of controlled processes altogether?

To test the influence of executive functions in repetitive timing in Experiment 1, executive load was controlled by manipulating the complexity of the spatial pattern of key presses. In the condition with low executive load (deterministic), participants were instructed to place the index, middle and ring fingers of the dominant hand on each of three adjacent keys and alternate between them in a regular sequence (i.e., index, middle, ring, index, middle, ring) and repeat this throughout the trial. The condition with high executive load (random) required the same three fingers to be depressed in a pseudorandom pattern. Pseudorandom generation is known to load strongly on executive control (Baddeley et al. 1998; Brown 1997; Holm et al. 2013; Miyake et al. 2000; Schneider et al. 2004). Importantly, the concurrent task (i.e., performance of the spatial sequence) was performed with the same effectors as the timing task, so that there was no confound between executive load and the number of effectors (i.e., inter-effector coordination). Task interference was indicated by the difference in timing variability between the two levels of cognitive load. Secondly, we compared temporal intervals below and above 1 s to test the extent to which executive functions are involved in the control of shorter and longer interval durations, the latter being predicted by the previous literature.

These predictions are based on the assumption that the secondary tasks (i.e., the generation of a repeating sequence in deterministic and a pseudorandom pattern in random) are performed equally accurate across conditions, so that timing variability is not upheld at the cost of sacrificing pattern reproduction fidelity or decreasing executive load. Therefore, performance accuracy of the secondary task was assessed in terms of accuracy of sequence in the deterministic, and statistical independence (entropy) and repetitiveness in the random condition. Experiment 2 followed up on the results from Experiment 1 by replicating the effects for the short intervals with a simpler and less demanding motor task and another type of cognitive load.

## Experiment 1

### Methods

#### Participants

Sixty students (age *M* = 26.4, SD = 4.46; 31 males) from Umeå University recruited via poster announcements participated in the study for a monetary compensation equivalent to 8 euro. Fifty-nine participants were right-handed according to the self-report. The participants gave written informed consent prior to the study, which was conducted in accordance with the Code of Ethics of the World Medical Association (Declaration of Helsinki) and approved by the local ethics committee (Dnr 09-065Ö). Four participants displayed behavior that did not comply with the task instructions, and either tapped a single key in all conditions or produced pseudorandom output in all conditions. We removed the data of these four participants from further analysis. Another participant produced data that were three SD below average in the H2 measurement (explained below) and two SD below average with respect to the complexity measurement (also explained below) and were removed from further analysis. Including these outliers in our statistical analysis produced the same pattern of results, including statistically reliable effects of executive load on timing. However, using the results of the trimmed sample seems more appropriate since the outliers clearly did not carry out the tasks according to the instruction.

#### Materials

Responses were given by pressing keys on a Yamaha DX-7 musical keyboard connected through MIDI to a Roland MPU-401-compatible MIDI interface connected to the computer ISA bus. Stimulus intervals were defined by the inter-onset interval of sampled percussion instrument sounds from a Kawai sound module. The synchronization sequence sounds had a cowbell character with sharp attack and relatively fast decay and a duration of approximately 80 ms. Feedback sound from pressing a key was sampled from a pair of claves, and this sound also had a duration of about 80 ms. The sounds were presented through loudspeakers with a sound pressure level (SPL) of 85 dBA at participant distance. A computer running the E-prime software package (Psychology Software Tools, Ltd) using the Windows 2000 operating system controlled computer screen instruction and triggered the onset of trials. Custom-designed software on a separate computer was used to control sound stimulus presentation and recorded participants’ responses. The sound presentation and recording software was run on a PC with the FreeDOS real-time operating system and communicated via MIDI with the response and stimulus devices.

#### Experimental procedure

All participants were tested individually. The participant was seated upright on a chair in front of the synthesizer keyboard and instructed to press three keys (C, D and E) on the keyboard using their dominant hand index, middle and ring fingers, at the same rate as the sounds presented from the loudspeaker. Additionally, the participant was instructed to press the keys according to either a repeating sequence (deterministic; C–D–E–C–D–E…) or to press the keys in a random order (random) without pressing the same key twice in a row. After 16 sound pulses, the participant continued to make key presses with the same interval and pattern as when synchronizing to the sounds. Responses were counted by the software, and each trial was terminated after 55 intervals (i.e., 56 key presses). The instructions stressed that it was important to avoid keeping time by rhythmically moving some other part of the body, such as the head or feet.

The inter-onset intervals (IOI) of the stimulus sounds were 524, 733, 1024 and 1431 ms. The length of the production phase of each trial was fixed at 71 responses. Each combination of the four IOIs and the two levels of executive load was replicated once, giving a total of 16 experimental trials. The experiment proper was preceded by a practice trial which was not included in the analysis. The order of executive load conditions was organized in identical blocks with either deterministic or random trials, consisting of four trials with rising or falling IOIs (e.g., 524,…, 1431 ms or 1431, …, 524 ms), in order to maintain equal spacing of IOI trial repetitions across participants. Starting block (deterministic or random) was counterbalanced across participants. Executive load condition alternated between blocks.

### Statistical analyses

To estimate randomness in the random key press sequences, we computed the normalized entropy of the distribution of pairs and triplets of consecutive key presses (bigrams and trigrams) according to:

1 $$H_{{}} = - \frac{{\mathop \sum \nolimits_{i = 1}^{n} p(x_{i} )\log \left( {p\left( {x_{i} } \right)} \right)}}{{ - \log \left( {\frac{1}{n}} \right)}}$$

where the relative frequency of all possible non-repeating key pairs or triplets *x* distributed across six legal pairs, and twelve legal key triplets, respectively, is used as a proxy for the probability of *x*, and the total number of different pairs or triplets is *n*. Notice that the normalization is done with the maximum entropy (uniform distribution) in the equation. For *n* = 6 (i.e., key pairs), the normalized entropy takes values in the range between 0.39 when the participant always alternates between the same two keys, with 1 meaning the participant distributed his or her key presses uniformly randomly between pairs of key sequences (see also Baddeley et al. 1998). Zero frequencies of observations were replaced with 0.01 before computing the entropy.

Randomness seems difficult for humans to achieve: We tend to structure our actions somewhat when requested to perform random actions (Baddeley 1966; Baddeley et al. 1998; Jahanshahi et al. 2000). The presence of a highly dependent structure would suggest that a participant did not comply with the random generation task, and degrees of structure across experimental conditions may indicate the varying impact on executive functions from those conditions. The information-theoretic measurements described above are limited by sequence length, because the number of observations required for reliable estimates of entropy grows exponentially with sequence length. One solution to the problem of assessing sequential structure in relatively small sequences is to assess repetitiveness in the sequence. This property is captured by the Kolmogorov complexity (Kaspar and Schuster 1987; Lempel and Ziv 1976), estimated by Faul’s (2005) MATLAB implementation. This measure reflects the algorithmic complexity required to reproduce, for example, a sequence of symbols. The quantity is not computable, but there are useful approximations, for example, from Kaspar and Schuster (1987), which produce easily computable estimates. A lower Kolmogorov complexity represents a lower randomness in the time series. To illustrate one of the benefits of including the Kolmogorov complexity, consider a fairly simple sequence of keys such as “C–D–E–D–C–E.” Repetitions of this sequence produces a H2 score of 1 (uniform distribution across all pairs) and a fairly respectable H3 value of 0.79 (c.f., empirical results below). However, the Kolmogorov complexity estimate of this sequence when repeated until the end of a production trial (i.e., after 55 key presses) would only be 0.59, whereas the expectation of a uniform random independent sequence of length 55 is about 1.4.

 For the deterministic condition, we computed the normalized Levenshtein distance (Levenshtein 1966) to determine performance accuracy. The Levenshtein distance is the number of basic operations (single element insertions, deletions and substitutions) needed to transform an observed string into a target string. The measure is normalized to the length of the observed string and hence varies from 0 for identical strings to 1 for strings with no sequential similarity. The measure is more sensitive than for example, string comparison because a simple shift of one position in one of the strings with respect to the other might render a string comparison method, suggesting no similarity between the strings at all, even though the strings are identical otherwise. For instance, the normalized Levenshtein distance between strings “C–D–E–D–C–E” and “D–E–D–C–E–C” is 1/3.

Continuation interval production variability was computed using data from the continuation phase in each trial. Inter-response intervals (IRIs) below 0.5 IOI or above 1.5 IOI were replaced by a moving average of seven intervals, centered on, but disregarding, the outlier. That is, the average of the three productions preceding and succeeding the outlier was computed and used to replace the outlier. A total of 1.0% of the interval productions were replaced in this fashion, mainly because of high interval variability right after the end of the synchronization phase.

The key-dependent measure in this study is timing variability. Because we compare interval productions across a wide range of IOIs, and the expected variability scales with interval duration, we employ the coefficient of variation (i.e., SD/mean) in the statistical analysis of variability. Moreover, to make the present results comparable to Holm et al. (2013), we also computed a timing variability measure based on next-to-adjacent intervals. This measure reduces drift impact on the variability estimates as it differentiates the time series. We denote this quantity local, and it is defined in Eq. 2.

2 $${\text{Local}} = \frac{100}{{\overline{x} }}\sqrt {\frac{{\mathop \sum \nolimits_{1}^{N - 2} \left( {x_{i + 2} - x_{i} } \right)^{2} }}{{2\left( {N - 2} \right)}}}$$

where *x* is an interval production and *N* is the number of productions.

### Results and discussion

#### Concurrent task performance

In deterministic, the accuracy of performance was operationalized as the Levenshtein distance between the produced sequence and the target sequence. The mean normalized Levenshtein distance and its SD were 0.012 ± 0.027, 0.010 ± 0.023, 0.011 ± 0.025 and 0.0076 ± 0.011 for target IOIs of 524, 733, 1024 and 1431 ms, respectively. A repeated-measures ANOVA with normalized Levenshtein distance as dependent variable and IOI as repeated-measures factor indicated no significant effect of ISI, *F*_3, 162_ = 0.75, *p* = 0.52. Thus, the participants performed the deterministic concurrent task with high consistency at all IOIs.

The randomness of the produced sequences in the random condition was estimated using the entropy of both produced bigrams and trigrams, as well as the Kolmogorov complexity of the sequence. If participants distributed their non-repeating key presses independently, then the expectation over participants and trials should be close to equally frequent sequences of three unique keys (“A–B–C”), as alternating keys (i.e., “A–B–A”). As seen in Table 1, there is a substantial bias to make sequences of three different key presses (“A–B–C”). This might reflect an influence from the deterministic condition or alternatively an innate bias for producing such sequences and suggest participants typically struggled with maintaining randomness in their key selections.

**Table 1:** Relative frequency of key triplets (ABC and ABA) and repetition errors (AA)

IOI	A–B–C	A–B–A	A–A
524	0.54	0.34	0.12
733	0.57	0.34	0.10
1025	0.55	0.33	0.12
1431	0.58	0.33	0.08

The bigram entropy was 0.91 ± 0.078, 0.93 ± 0.081, 0.92 ± 0.12 and 0.93 ± 0.085 for target IOIs of 524, 733, 1024 and 1431 ms, respectively. In a repeated-measures ANOVA with entropy as dependent variable and IOI as repeated-measures factor, there was no effect of IOI, *F*_3, 162_ = 2.18, *p* = 0.09. The trigram entropy yielded 0.82 ± 0.019, 0.88 ± 0.017, 0.87 ± 0.19 and 0.88 ± 0.018 for IOIs of 524, 733, 1024 and 1431 ms, respectively. In a repeated-measures ANOVA with entropy as dependent variable and IOI as repeated-measures factor, there was an effect of IOI, *F*_3, 162_ = 8.79, *p* < 0.001. Contrast tests showed that the entropy for ISI = 524 ms was reliably lower than any of the other ISI conditions.

The Kolmogorov complexity was 1.26 ± 0.31, 1.26 ± 0.32, 1.25 ± 0.35 and 1.27 ± 0.33 for IOIs of 524, 733, 1024 and 1431 ms, respectively. A repeated-measures ANOVA demonstrated no significant effect of IOI on Kolmogorov complexity, *F*_3, 162_ = 0.22, *p* = 0.88. Taken together, the randomness analyses suggest some decrease in randomness at 524-ms IOI.

#### Timing performance

As expected, the mean IRIs closely follow the IOI and intra-trial variability in mean IRI is rather small, as seen in Table 2. Moreover, there appears to be no significant difference in mean IRI between the deterministic and random conditions. This impression was supported by an ANOVA with mean IRI as dependent variable and IOI and executive load as repeated-measures factors, showing no main effect of condition, *F*_1,__54_ = 0.12, *p* = 0.91, a significant effect of IOI, *F*_3,__162_ = 2789, *p* < 0.00001, and no significant IOI × executive load interaction, *F*_3, 162_ = 1.27, *p* = 0.29.

**Table 2:** Mean IRI and inter-trial SD for executive load and IOI conditions across replications and participants

	524 ms		733 ms		1024 ms		1431 ms	
	*M*	SD	*M*	SD	*M*	SD	*M*	SD
Random	514.8	44.2	708.6	57.1	996.1	77.7	1380.0	122.1
Deterministic	511.9	35.1	711.9	53.7	987.2	75.3	1390.3	120.9

**Fig. 1:**
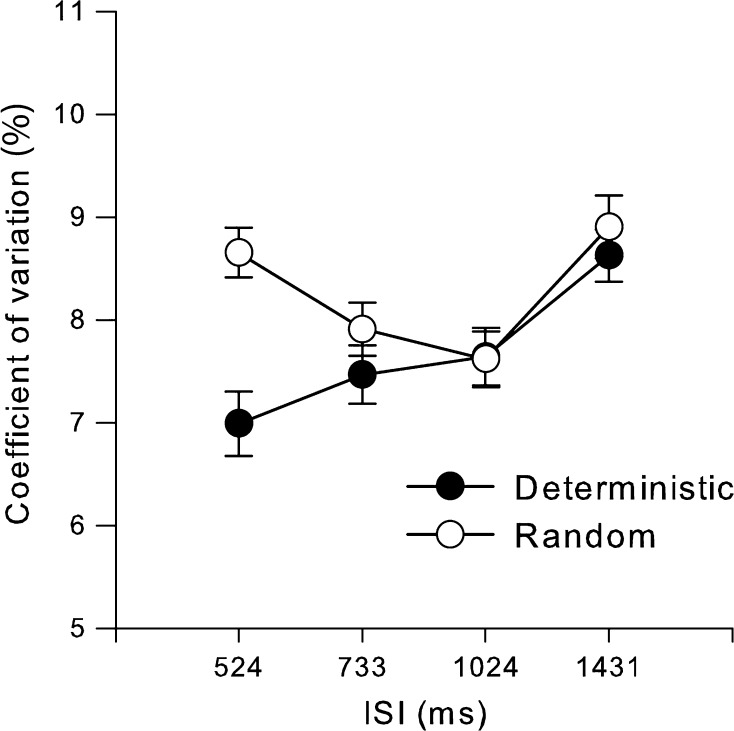
Average coefficient of variation as a function of executive load (separate lines for deterministic and random conditions) and inter-stimulus interval. *Error bars* are 1 SEM

 We now address the two main hypotheses, i.e., that timing variability be higher with greater executive load and that this effect be more pronounced for longer intervals. We used the coefficient of variation of intra-trial SD, for comparable estimates across IOIs, based on the 55 produced intervals, as displayed in Fig. 1. A 2 executive load × 4 IOI repeated-measures ANOVA exhibited significant main effects of executive load, *F*_1_, _54_ = 7.10, *p* = 0.01, and of ISI, *F*_3, 162_ = 10.90, *p* < 0.001, and of their interaction, *F*_3_, _162_ = 5.28, *p* = 0.002. As seen in all Fig. 1, the interaction resides in more pronounced effects for load of the shorter ISIs and no effects at all above 1 s.

**Fig. 2:**
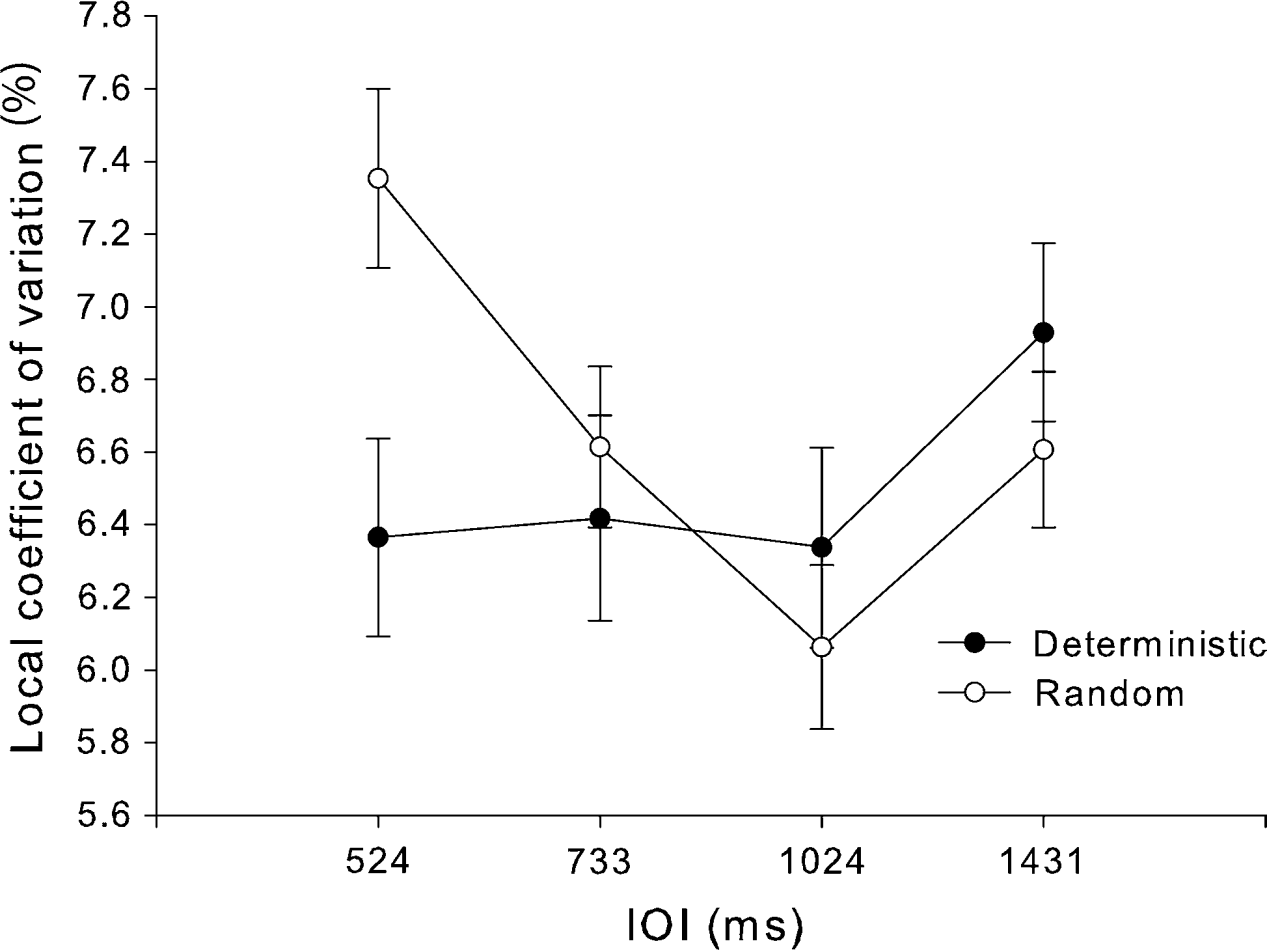
Average local coefficient of variation as a function of executive load (separate lines for deterministic and random conditions) and inter-stimulus interval. *Error bars* are 1 SEM

To make the present findings comparable to Holm et al. (2013), we also computed the local coefficient of variation for the interval productions. The results are presented in Fig. 2. As seen, there appears to be an interaction between executive load and IOI such that executive load increases the local coefficient of variation at 524-ms IOI, but has no impact on the other IOI conditions. We tested the effects of executive load and IOI in a 2 executive load × 4 IOI repeated-measures ANOVA. There was no significant effect of executive load, *F*_1, 54_ = 0.61, *p* = 0.44, a significant effect of IOI, *F*_3, 162_ = 5.52, *p* < 0.001, and a significant interaction, *F*_3_, _162_ = 6.81, *p* < 0.001. Contrast tests of the interaction showed only that the executive load difference at 524-ms IOI ms was reliably larger than at any other IOI, *F*_1, 54_ > 3.7, *p* < 0.05.

Experiment 1 showed that variability was higher in random than in deterministic, suggesting that executive functions influence sustained performance of motor timing tasks. Moreover, the effect of executive load was substantially larger for the shortest interval used, indicating that executive functions are involved in sub-second repetitive motor timing. This appears to differ from Holm et al. (2013), who suggested that their small but nonsignificant effect of executive control was mediated through motor coordination. The greater statistical power enabled by having more than twice as many participants in Experiment 1 may explain this difference. Notably, the magnitude of the timing variability as well as other aspects of the performance was almost identical to those of Holm et al. (2013), indicating that similar conditions applied in all other respects.

There was not only an effect of executive load in short IOIs, but it was also strongest in the shortest IOI, counter to the general prediction regarding load and IOI. One possible explanation is that the generation of pseudorandom responses in itself takes a certain amount of time. If the time available between 733-ms taps is just sufficient, on average, thwarted performance on 524-ms IOI tapping may explain this pattern of results. For instance, Baddeley (1966) found that redundancy increased linearly with response rate in a random letter generation task from 4 to 0.5 s IRI. Moreover, if random generation is time demanding, one might expect two types of responses when time is constrained. The first is worse randomization performance, i.e., less randomness, and the other is that participants prioritize finishing the cognitive task by increasing the inter-response intervals (IRI), as they are free to do so during the production phase. The IRI was, however, unaffected by executive load, but at least one measure of randomness provided evidence that key-tapping sequences were least random in the shortest interval production condition. Another possible explanation is that the complexity of coordinating three fingers constitutes a cognitive load in itself. Regardless of this potential load confound with IOI, Experiment 1 suggests that there may be an effect of executive load on sub-second motor timing variability.

## Experiment 2

The novel finding from Experiment 1 was that at least the executive function aspect of cognitive control is involved in sub-second repetitive motor timing, as demonstrated by the reliable effect of load at 524 ms. It was, however, complicated by the reversed difference between 524 and 733 ms, relative expectations, the finding that randomness was slightly decreased for 524 ms, and the suspicion of confounds between cognitive load, IOI and motor complexity load. Experiment 1 employed a fairly complex timing response method in that patterns of digit pressing were required to indicate timing. This may increase motor or coordination noise in the timing responses. Ideally, the effectors used for timing measurement should contribute as little error as possible to make the timing component more visible.

Experiment 2 was designed to address these outstanding factors by (1) using a simpler motor task and (2) controlling the experienced cognitive load through pupil dilation eye. This was done for the relevant shorter IOIs only, because effects for longer IOIs are already established.

Beating a drumstick is found to yield less variable timing performance than finger tapping (Madison et al. 2013). An indicator of the amount of working memory load is pupil dilation and blink rate (Kahneman and Beatty 1966; Karatekin et al. 2004; Siegle et al. 2008). Specifically, higher working memory load is associated with higher mean and peak pupil dilation as well as an increased blink rate. These physiological variables may also be easily and un-intrusively recorded, rendering it a reliable means of validating working memory load.

The use of a drumstick to indicate timing made it difficult to include the cognitive task in the motor performance. Instead, we employed a Sternberg letter memory task to test the influence of working memory, while participants produced isochronous intervals. The Sternberg task involves a brief presentation of a set of items (e.g., letters) to be retained during a delay period. After this, an item is presented and the participants decide whether the item was present in the original set. The larger the set, the harder the task.

Specifically, this design allowed us to make working memory load independent of base interval duration and the effect of load constant across the entire period of measurement. Finally, we measured working memory load independently by monitoring pupil dilation and blinks.

### Methods

#### Participants

Twenty-five students (age *M* = 25.5, SD = 4.69; 12 males) from Umeå University recruited via poster announcements participated in the study for a monetary compensation equivalent to 8 euro. Twenty-three participants were right-handed by self-report. The participants gave written informed consent prior to the study, which was conducted in accordance with the Code of Ethics of the World Medical Association (Declaration of Helsinki) and approved by the local ethics committee (Dnr 09-065Ö).

#### Materials

Metronome sound stimuli consisted of 50-ms 262 Hz sinewave tones played through loudspeakers in front of the participant. Timing responses were made by hitting a drum pad with a drumstick. The only feedback sound was the unamplified noise produced when the drumstick hit the drum pad. The drum pad contained a piezoelectric element connected to an Arduino Uno board, which recorded the beats based on an in-house developed algorithm. Specifically, the Arduino sampled electric signals at 1 kHz. Beats were defined by a threshold on the discrete approximate derivative of the piezoelectric signal. Timing properties of the Arduino setup were measured with a National Instruments USB 6211 sampling at 10 kHz. It indicated a timing error (SD) of 1.48 ms. Recorded beats were transferred to a PC running Windows 7 via the TCP/IP protocol. The PC ran in-house developed MATLAB (MATLAB 2010, 32 bit) scripts including some PTB 3 library functions (Brainard 1997) to present letter strings and record Sternberg task responses. Letter strings were presented on a 24″ monitor. A desk-mounted EyeLink 1000 was used to record eye movements and pupil dilation, sampling at 500 Hz.

#### Procedure

Individually tested participants were seated in front of the monitor and received written instructions for the test. They were then asked to lean their heads in a chin rest, positioning their eyes about 75 cm from the monitor. A drum pad was then placed so that the participant could comfortably hit it with a drumstick using their dominant hand. The non-dominant hand rested on the computer keyboard in front of the participant and was used to indicate responses in the Sternberg task. The eye tracker was then calibrated. The experimental trials were organized such that the participant first listened to a metronome playing sine tones in loudspeakers in front of the participant and synchronized to the sounds for 15 beats. The string of consonants was then presented on the monitor for 1 s. The letters were centered on the monitor in white on black in “Arial” style and font size 40. The strings were about 2.4 (three letters) and 5.6 (seven letters) degrees across at participant distance. The monitor then went blank, and the metronome was switched off. Participants continued to produce the intervals unsupported for 6285 ms, when a letter probe presented on the monitor indicating them to stop producing intervals. The letter was from the initially presented letter string in 50% of the trials. Participants responded whether the letter probe was from the string or not using the keyboard. Participants were instructed to maintain fixation in the middle of the screen throughout each trial. Two different inter-stimulus onsets (IOI) were used in the metronome, 524 and 733 ms, respectively. Each IOI by letter string length condition was repeated 40 times for a total of 160 trials per participant. IOI and letter string conditions were randomly mixed for each participant.

#### Results

##### Sternberg task performance

Average proportion of correct responses on the Sternberg task was *M* = 0.94 and 0.76 in the 524-ms IOI for 3 and 7 letter conditions, respectively. The average proportion of correct responses in the 733-ms IOI conditions was *M* = 0.95 and 0.73 for 3 and 7 letter conditions, respectively. Thus, working memory load (i.e., number of letters) appears to have impacted on performance, but not IOI. This observation was qualified by submitting the results to a 2(working memory load) × 2(IOI) repeated-measures ANOVA. It showed a significant main effect of working memory load, *F*_1, 24_ = 119.4, *p* < 0.0001, but not of IOI, *F*_1, 24_ = 0.33, *p* = 0.57. The interaction was not significant, F_1, 24_ = 3.26, *p* = 0.084.

##### Eye movement results

**Fig. 3:**
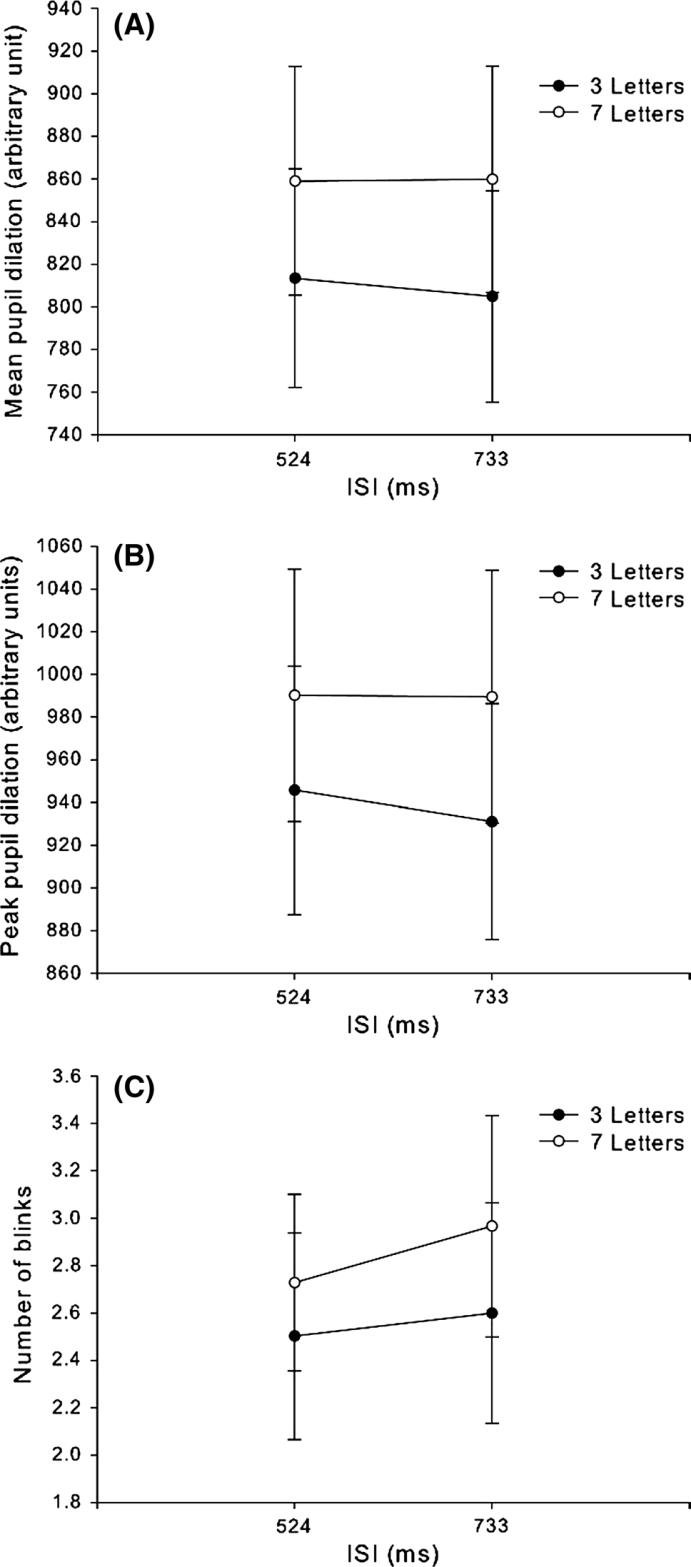
Eye indicators of executive load. a shows peak pupil dilation, b shows mean pupil dilation, and c shows number of blinks as a function of ISI and executive load (separate lines for 3 and 7 letters conditions). *Error bars* are 1 SEM

The average and peak pupil dilations as well as number of blinks from the interval production period are summarized in Fig. 3. The number of letters but not IOI appears to have impacted on pupil dilation and number of blinks. All eye movement data were submitted to a 2(working memory load) × 2(IOI) repeated-measures ANOVA. There was a significant main effect of working memory load *F*_1, 24_ = 23.2, *p* < 0.001, no significant main effect of IOI, *F*_1, 24_ = 0.23, *p* = 64, and no significant interaction, *F*_1, 24_ = 0.28, *p* = 0.60. Similarly, peak pupil dilation (panel B in Fig. 2) showed a significant main effect of working memory load, *F*_1, 24_ = 24.9, *p* < 0.0001, no main effect of IOI, *F*_1, 24_ = 0.76, *p* = 0.39, and no significant interaction, *F*_1, 24_ = 0.56, *p* = 0.46. Two outliers with strange blink recordings were removed from further analysis because they both displayed one order of magnitude more blinks than the third most blinking participant. In the reduced sample (summarized in panel C of Fig. 2), there was a significant main effect of working memory load, *F*_1, 22_ = 5.67, *p* = 0.026, but not of IOI, *F*_1,22_ = 1.32, *p* = 0.26. There was also no significant interaction, *F*_1, 22_ = 0.36, *p* = 0.55.

##### Timing performance

The within-trial average IRI and SD of interval productions are summarized in Table 3.

**Table 3:** Mean IRI and inter-trial SD for executive load and IOI conditions across replications and participants

	524 ms		733 ms	
	*M*	SD	*M*	SD
7 Letters	525.8	28.1	712.3	39.2
3 Letters	523.3	26.0	717.1	34.4

**Fig. 4:**
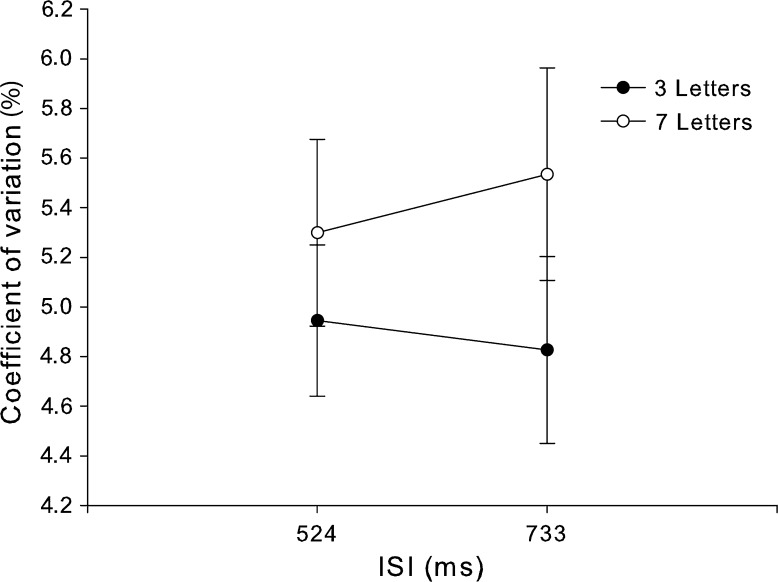
Average coefficient of variation as a function of executive load (separate lines for 3 and 7 letter conditions) and inter-stimulus interval. *Error bars* are 1 SEM

As in Experiment 1, the coefficient of variation was used as measure of timing performance. The averages are summarized in Fig. 4. As predicted, given the simpler timing task of Experiment 2, the overall timing variability was lower than in Experiment 1. There seems to be a clear effect of working memory load and potentially an interaction with IOI. This observation was partly supported by the ANOVA results, which showed a significant main effect of working memory load at *F*_1, 24_ = 22.1, *p* < 0.0001 but no main effect of IOI, *F*_1, 24_ = 0.13, *p* = 0.72. Furthermore, the interaction was not significant, *F*_1, 24_ = 3.15, *p* = 0.089.

The working memory load imposed by the Sternberg task clearly affected task performance such that the longer letter string conditions lead to lower letter recognition performance. Furthermore, all eye movement measurements were in agreement with a working memory load effect––pupil mean and peak dilation as well as blink rate were all reliably higher in the high load conditions. Furthermore, none of the measurements indicated any effects of IOI nor any interaction.

### General discussion

The main purpose of this study was to test whether cognitive control is involved in repetitive motor timing below 1 s. In Experiment 1, the primary task (i.e., motor timing) and the secondary task (sequence generation) were both performed with the same effector. Results confirmed an effect of executive control in IRIs below 1 s. Experiment 2 replicated an effect on sub-second motor timing, this time for working memory load. Taken together, these findings strongly indicate that cognitive control is involved in motor timing in the sub-second range.

Demonstrating this using two complementary experiments is important, because dual-task designs involving timing performance tend to be methodologically challenging. The studied cognitive processes should ideally be independent of the effector system used for performance. Using several effectors simultaneously introduces a motor coordination requirement that is not present when the tasks are carried out separately. Therefore, if two cognitive tasks are tested using two different effector systems, and concurrent performance indicates interference, then the interference can have at least two sources: (1) shared and limited cognitive mechanisms involved or (2) increased motor coordination requirements. Previous dual-task studies of timing have not always acknowledged this confound. For instance, Kee et al. (1986) only found timing interference when solutions to the simultaneous anagram task were spoken *aloud* and not when they were read out in silence. Holm et al. (2013) found no reliable evidence for timing interference from executive load when only one hand was used to perform a rhythmic task, but a significant effect when the two hands were used together in the high executive load condition. One of the conclusions of that study was that effects of executive functions on timing performance may reflect control of effector coordination rather than timing itself. Similarly, a recent study by Maes et al. (2015) found that cellists increased tempo variability when they concurrently performed a digit-counting task. Thus, although the different concurrent tasks and motor responses in the present study make direct comparisons difficult, the critical question whether both executive functions and working memory affect sub-second motor timing was affirmed across the experiments.

Notably, neuroimaging data suggest that timing tasks load more on cognitive control as they become sensory rather than motor, discrete rather than repetitive, and use supra-second rather than sub-second durations (Lewis and Miall 2003; Rammsayer and Troche 2014). The repetitive sub-second motor timing task used in the present experiments would thus belong to a class of timing tasks that has comparatively low control involvement, but we nevertheless find evidence for a role for cognitive control. This suggests that cognitive control may have some effects on the performance of most, if not all, explicit timing tasks, even if the strength of the effect probably varies with task characteristics. Thus, the dichotomist view of automated versus controlled processes across some interval duration around 1 s may be replaced with the view of diminishing but present cognitive control well below 1 s of interval productions.

The present results bear on the long established relationship between timing and intelligence. In general, intelligence is positively correlated with accuracy of performance in a wide range of tasks that involve perceptual or motor processing of temporal information (Jensen 2006). This has also been demonstrated for the isochronous generation task used in the present study (Holm et al. 2011; Ullén et al. 2015). Earlier analyses indicate that this relation is in part due to bottom-up mechanisms that are independent of cognitive control (Holm et al. 2011; Madison et al. 2009; Ullén et al. 2008) and may result from, for example, neural timing properties associated with prefrontal white matter regional volume (Ullén et al. 2008). For instance, the continuation timing × IQ relation is unaffected by experimental manipulation of state motivation to perform well (Ullén et al. 2012).

The present findings suggest that top-down influences might also contribute to correlations between intelligence and motor timing. Such influence could operate in several different ways, for example, by challenging the participants’ highest level of performance, thus exposing the surplus capacity when cognitive resources are almost depleted. The present level of knowledge cannot assess this and other alternative scenarios, and further research is required to more closely map the nature of the timing–IQ relationship.

The presence of cognitive control also in periods of only 0.5 s seems to suggest that it is predictive control demands, rather than the time itself, that determine the involvement of cognitive control. First, timing is intrinsically predictive because determining when to act upon a future event requires the ability to predict it. As the time frame of a planned action increases, predictive uncertainty reasonably increases too, because more uncertain things may happen in a longer period of time. For instance, signals representing longer time periods run a greater risk of interference or decay. Holm and Madison have argued before (Holm and Madison 2013) that cognitive control mechanisms may constitute a general-purpose resource for controlling timed behavior that is recruited in response to the computational demand. Importantly, under this perspective, it is not the time frame itself that should determine whether general purpose and computationally expensive involvement of cognitive control, but rather the computational demands invoked in guiding behavior accurately. Conceivably, the general purpose of cognitive control for timing also comes at the price of less time-efficient interventions compared to dedicated timers closer to the sensory or motor levels. A straightforward prediction is then that the influence of cognitive control should increase with duration. While the present study was limited to testing for cognitive control at sub-second motor timing, and the results of Experiment 1 did not display the expected increase in effect with IOI, the wider pattern of results from previous studies certainly support this idea. A general conclusion from the present study is therefore that there exists a functional basis for a top-down relationship between cognition and timing, at least under similar conditions to those in these experiments.
